# Estimating bacterial load in *S. aureus* and *E. coli* bacteremia using bacterial growth graph from the continuous monitoring blood culture system

**DOI:** 10.1007/s10096-024-04893-w

**Published:** 2024-07-29

**Authors:** Leehe Turkeltaub, Livnat Kashat, Marc V. Assous, Karen Adler, Maskit Bar-Meir

**Affiliations:** 1 Pediatric Department, Shaare-Zedek Medical Center, Jerusalem, Israel; 2 The Microbiology Laboratory, Shaare-Zedek Medical Center, Jerusalem, Israel; 3 Pediatric Infectious Diseases, Shaare-Zedek Medical Center, Jerusalem, Israel; 4 The Faculty of Medicine, The Hebrew University, Jerusalem, Israel

**Keywords:** Blood culture, Bacterial load, *S. Aureus*, *E. Coli*, Outcome

## Abstract

**Background:**

We examined whether the time to positivity (TTP) and growth and detection plot graph (GDPG) created by the automated blood culture system can be used to determine the bacterial load in bacteremic patients and its potential association correlation with disease severity.

**Methods:**

Known bacterial inocula were injected into the blood culture bottles. The GDPGs for the specific inocula were downloaded and plotted. A cohort of 30 consecutive clinical cultures positive for *S. aureus* and *E. coli* was identified. Bacterial load was determined by comparing the GDPG with the “standard” curves. Variables associated with disease severity were compared across 3 bacterial load categories (< 100, 100–1000, > 1000 CFU/mL).

**Results:**

*S. aureus* growth was sensitive to the blood volume obtained whereas *E. coli* growth was less so. A 12-hour delay in sample transfer to the microbiology laboratory resulted in a decrease in TTP by 2–3 h. Mean TTP was 15 and 10 h for *S. aureus* and *E. coli*, respectively, which correlates with > 1000 CFU/mL and 500–1000 CFU/ml. For *S. aureus*, patients with a bacterial load > 100 CFU/mL had a higher mortality rate, (OR for death = 9.7, 95% CI 1.6–59, *p* = 0.01). Bacterial load > 1000 CFU/mL had an odds ratio of 6.4 (95% CI1.2-35, *p* = 0.03) to predict an endovascular source. For *E. coli* bacteremia, we did not find any correlations with disease severity.

**Conclusion:**

GDPG retrieved from the automated blood culture system can be used to estimate bacterial load. *S.aureus* bacterial load, but not *E.coli*, was associated with clinical outcome.

**Supplementary Information:**

The online version contains supplementary material available at 10.1007/s10096-024-04893-w.

## Background

Blood cultures remain the gold standard for diagnosing sepsis and bacteremia, even in the era of molecular-based techniques. Bacterial species, susceptibility profile, and the site of infection are the main factors that clinicians consider when determining the patient’s optimal therapy. The magnitude of the bacterial load is usually unknown and can be indirectly estimated from the “time to positivity” (TTP); that is, the time elapsed between the inoculation of blood into the blood culture bottles and the time of growth detection by the automated blood culture system.

The concept that greater bacterial burden is associated with worse clinical outcome is not new. Quantitative blood culture studies in adults in the 1930s and 1950s and in children in the 1970s have shown that higher pneumococcal burden in the blood was associated with poor clinical outcome [1]. More recently, studies that have used TTP or bacterial DNA load showed an association between higher bacterial load and mortality or poor outcome in *Neisseria meningitidis* [2], *Staphylococcus aureus* [3, 4], methicillin-resistant *S. aureus* (MRSA) [5] and *Streptococcus pneumoniae* bacteremia [6].

The automated blood culture system responds to the change in CO_2_ concentration due to bacterial growth. Each blood culture vial contains a sensor, that is monitored every 10 min, for either an increase in fluorescence or a change in color (depending on the manufacturer), which are proportional to the amount of CO_2_ produced by the growing bacteria. Once the system detects the exponential growth phase, a “positive” signal is produced. The data for each vial is saved as a growth and detection plot. Classically, the growth of bacterial culture starts with a lag phase and continues with logarithmic and exponential phase [7]. If the lag phase is relatively insignificant, then the TTP can be used as a reliable estimate of the bacterial load in the blood. Moreover, perhaps the shape of the growth and detection plot graph (GDPG) created by the automated blood culture system can be used for assessing the initial bacterial load in the patient’s blood.

We aimed to examine whether the TTP and GDPG can be used to determine the initial bacterial load in the blood among patients with *S. aureus* and *E. coli* bacteremia and whether there is a correlation between the bacterial load and clinical outcomes associated with disease severity.

## Methods

### Bacterial counts

Wild-type strains of *E. coli* (ATCC 25,922) and *S. aureus* (ATCC 25,923) were used to determine the bacterial generation time and TTP for different bacterial loads. CFU/mL was calculated from 1 McFarland suspension for each bacterial strain, by serially diluting the suspension in a 96-well plate, plating 3 10 ηL drops of each dilution on a blood agar plate, and averaging the number of colonies in each spot (Figure S1). This technique was used for all bacterial counts detailed in this work. A triplicate of experiments estimated that 1 McF is equal to 1.5 × 10^8^ CFU/ml of *E. coli* ATCC 25,922 and 2.63 × 10^8^ CFU/ml of *S. aureus* ATCC 25,923, respectively. For each experiment, the specific bacterial load was calculated and diluted from the 1 McF suspension to result in three 1 ml suspensions at concentrations of 55.56 CFU/ml, 5.56 CFU/ml, and 0.56 CFU/ml (supplement 1). These were added to 9 ml sterile saline (so as to create solutions containing approximately 500, 50, and 5 bacteria respectively), and 9 ml of this final suspension was then injected into the aerobic bottle (BACTEC™ FX blood culture system. Becton, Dickinson and Co., NJ).

### Determining the correlation between TTP and bacterial load

To examine the correlation between the bacterial load and TTP, and thereby indirectly assess whether the lag phase plays a significant role in the growth pattern of bacteria in the blood culture bottles, duplicates of *S. aureus* and *E. coli* bacterial concentrations of 500, 50 and 5 CFU were prepared and inoculated into blood culture bottles, that were then incubated in the BACTEC FX incubator. Time to detection in hours was recorded for each concentration. Then, culture bottles were incubated for 12 and 18 h for *S. aureus* and 6 and 12 h for *E. coli*. The CFU/mL was counted at the end of incubation. The multiplication factor (MF) of the bacteria was calculated by dividing the growth at time point 2 by time point 1, and the number of multiplications in 6 h was calculated by log (MF)/log (2).

### The Growth & detection plot graph (GDPG) for different bacterial loads

Again, known bacterial inocula were injected into the blood culture bottles. The GDPGs for the specific inocula were downloaded from the BACTEC™ FX database (epicenter ™ software) and converted into Microsoft Excel format. The rate of fluorescence increase was calculated by dividing each cell in the Excel column representing fluorescence reading by the preceding cell. Then, we plotted the growth and detection graph with time in hours on the x-axis and the rate of fluorescence increase on the y-axis (Supplementary file 2). To verify that the growth kinetics of ATCC and clinical strains is not different we have inoculated 2 wild-type *E.coli* strains, 2 extended-spectrum beta-lactamase (ESBL) -producing strains and 2 tolerant strains, as well as 3 different concentrations of clinical strains. The growth kinetics of these strains was similar with TTP correlating with the concentration inoculated into the bottle (Supplementary file 2).

### Clinical growth curves

A cohort of 60 consecutive patients with *S. aureus* and *E. coli* bacteremia (30 for each bacterial species) were identified through the logs of the microbiology laboratory. The positive culture bottles were weighed to estimate the blood volume inoculated. Since the laboratory technicians are instructed to obtain 2 ml of blood for gram stain and plating, we calculated the blood volume inoculated into the bottle by adding 2 ml to the bottle weight. GDPGs were retrieved from the BACTEC™ FX database and converted into Microsoft Excel format. The growth curve was plotted as described above, and compared with the growth curves designed in the previous step. The estimated inoculum was determined for each patient in a semi-quantitative fashion, divided into three categories of bacterial load: low < 100 CFU/mL, medium 100–1000 CFU/mL and high > 1000 CFU/mL.

### Clinical data

The patients’ clinical data were collected from the medical charts. We recorded age, gender, time of blood draw, time at which the bottles were placed in the BACTEC™ FX incubator (“protocol start”), medical diagnoses in the current admission and co-morbidities, origin of bacteremia, extended bacteremia (defined as bacteremia of ≥ 2 days), length of hospital stay, days of fever and surrogate markers for disease severity including death, intensive care admission, and use of vasopressors and mechanical ventilation.

The Quick Sequential (sepsis-related) Organ Failure Assessment Score (qSOFA, range 0–3 points) and the Pitt bacteremia score (PBS, range 0–14) were calculated as well.

### Statistical analysis

Analysis was performed with SPSS V25.0 (SPSS, Chicago, IL USA). Continuous variables were compared with a t-test for normally distributed variables and the Mann–Whitney U-test otherwise. Categorical variables were compared with χ2 test. The correlation between TTP and inoculum was compared using Spearman’s rank correlation.

Variables associated with disease severity were compared between the bacterial load categories using analysis of variance (ANOVA). All reported p-values are two-sided and considered significant at *p* < 0.05.

### Sample size calculation

Sample size calculation was performed for length of stay [8, 9]. A cohort of 12 patients with *S. aureus* and 30 patients with *E. coli* bacteremia are required to detect a difference of 5 days of hospital stay with a power of 80% and a level of significance of 0.05.

### Ethics approval

The study was approved by the SZMC institutional review board (approval #0172 − 21). Informed consent was waived since no intervention was made and patient information was de-identified.

## Results

### Time to positivity correlates with bacterial load in the blood

Table 1 shows a direct correlation between TTP and the bacterial inoculum. This direct correlation suggests that the lag phase does not play a significant role in the bacterial growth pattern in the automated blood culture system, and therefore TTP can be used as an estimate of initial bacterial load. Table 2 shows the generation time for *E. coli* and *S. aureus*, as calculated between two-time points for the different concentrations. *E. coli* doubles every 18–24 min, whereas *S. aureus* requires 1–1.2 h to double.

**Table 1:** The correlation between time to positivity (TTP) and bacterial load for *E. Coli* and *S. Aureus*

Bacterial load, CFU^*^	*E. coli* average TTP in hours	*S. aureus* average TTP in hours
1000	9:30	17:10
500	10:20	18:40
50	11:30	23:10
5	13:20	26:50

*Colony-forming units per vial

**Table 2:** Generation time for *S. aureus* and *E. coli* for different bacterial loads

	CFU*	Growth point I (average)^**^	Growth point II (average) ^**^	Multiplication factor (MF = growth point 2/growth point 1)	Number of multiplications in 6 h, X [log multiplication factor/log(2)]^*^	Generation time, hours (6 h/X)^***^
S.aureus	500 50 5	5*10^4^±8*10^4^ 1.5*10^4^±2*10^4^ 1.3*10^3^±1.9*10^3^	3*10^6^±1.4*10^6^ 38*10^4^±40*10^4^ 4.7 × 10^4^ ± 2.3*10^4^	60 25 35	6 4.6 5	1 1.2 1.2
E.coli	500 50 5	3*10^3^±1.9*10^3^ 6.5*10^2^±6*10^2^ 1.3*10^3^±6*10^2^	1.4*10^8^±8*10^8^ 2*10^8^±9*10^7^ 3*10^7^±2*10^7^	4*10^5^ 3.5*10^5^ 2*10^4^	18 18 14	0.3 0.3 0.4

^*^CFU- Colony-forming units

^**^ Growth point I & II: for SA **12 & 18 h**, for EC **6 & 12 h**, respectively

^***^ Generation time is calculated from 2^x^ = multiplication factor; x = log (MF)/log (2)

^*^ Generation time is calculated from 2^x^ = multiplication factor; x = log (MF)/log (2)

^**^ Growth point I & II: for SA **12 & 18 h**, for EC **6 & 12 h**, respectively

*Technical characteristics of clinical blood cultures.* Sixty consecutive patients with *S. aureus* and *E. coli* bacteremia (30 in each group) were enrolled. The technical characteristics of blood cultures are shown in Table 3. The median time between the blood draw and the start of the incubation protocol (time of specimen transfer to the lab and insertion into the incubator) was 4 and 8 h for *S. aureus* and *E. coli*, respectively, and the median blood volume inoculated was 4.5 mL in the aerobic bottles and 3.2 mL in the anaerobic bottles. There was a significant correlation between the blood volume and the TTP (e.g. the estimated inoculum) only for *S. aureus* in the anaerobic bottle, but not in the aerobic bottles (Pearson correlation= -0.6, *p* = 0.04). No correlation was found between blood volume and *E. coli* TTP/inoculum in either bottle. To examine the effect of delay in the incubation of blood culture bottles, we again inoculated 50 and 500 CFU into a duplicate of bottles. One duplicate was incubated immediately, whereas the other one was left at room temperature for 12 h before entering the incubator. For *E. coli*, a 12-hour delay in incubation resulted in an average of 3.6 ± 0.5 shorter TTP and for *S. aureus* the delay resulted in an average of 2.2 ± 0.7 h shorter TTP. These results were similar between 50 and 500 CFU.

**Table 3:** Characteristics of blood cultures positive for *S. aureus* and *E. coli*

Variable	*S. aureus*	*E. coli*	*P* value
**Time elapsed between blood draw and incubation**, **hours** Median, range (SE)^1^	4.3, 0–20 (0.4)	8, 0–22 (0.4)	0.1
**Blood volume**,**aerobic bottle**, **ml** Median, range (SE)	4.5, 0.65–9.5 (0.4)	4.4, 1.4–11.6 (1)	0.6
**Time to positivity**, **aerobic bottle**, **hours** Median, range (SE)	14.2, 6–34 (0.4)	10.2, 3.4–18 (0.6)	0.4
**Blood volume**, **anaerobic bottle**, **ml** Median, range (SE)	2, 0.5–7.4 (0.6)	3.5,0.5–8.4 (0.4)	0.2
**Time to positivity**, **anaerobic bottle**, **hours** Median, range (SE)	12.7, 6–19 (0.6)	8.5,2.6–20.1 (0.4)	0.4

*Correlation between bacterial load and clinical outcomes.* Of the 30 pairs of culture bottles – one each of aerobic and anaerobic – *S. aureus* grew in both bottles in only 20% of sets and *E. coli* in 30% (6 and 9 full sets, respectively). In the other pairs, *S. aureus* grew preferentially in the aerobic bottle (23/30) whereas *E. coli* grew preferentially in the anaerobic bottle (24/30). For the pairs with 2 positive bottles, the difference in TTP between the bottles was < 1 h in 8/9 pairs for *E. coli* and 2/6 for *S. aureus*. When the difference was > 1 h, we used the mean TTP to estimate the bacterial load.

**Fig. 1:**
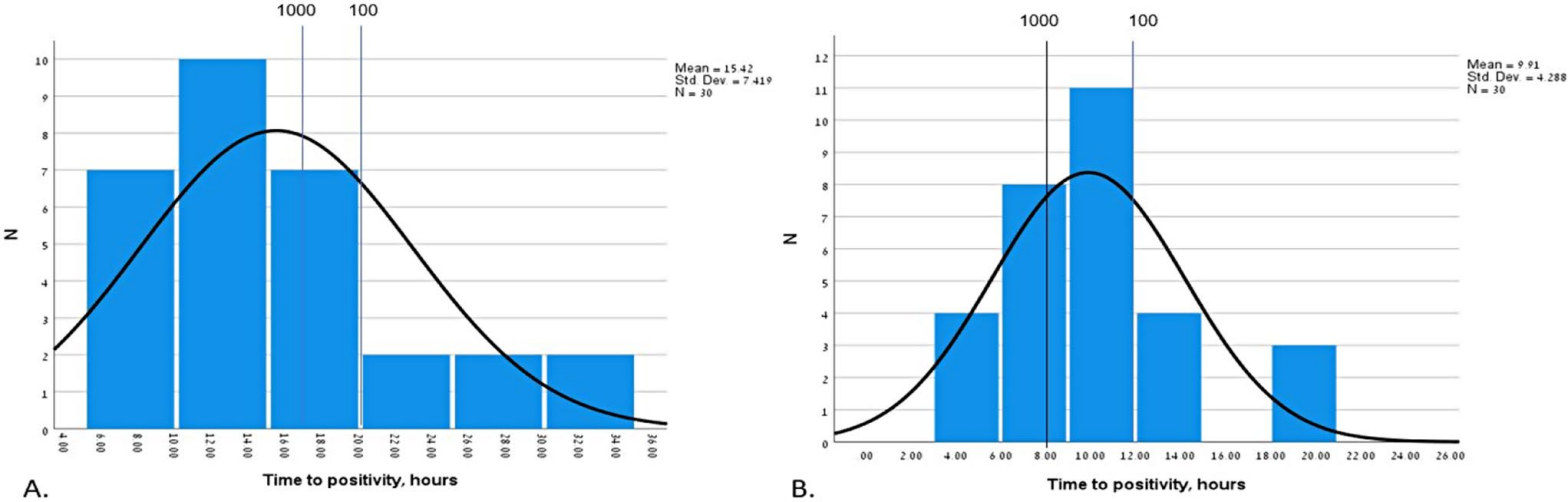
Histogram of Time to Positivity (TTP) in a cohort of 30 clinical blood cultures growing *S. aureus* (**A**) and *E. coli* (**B**)

**Fig. 2:**
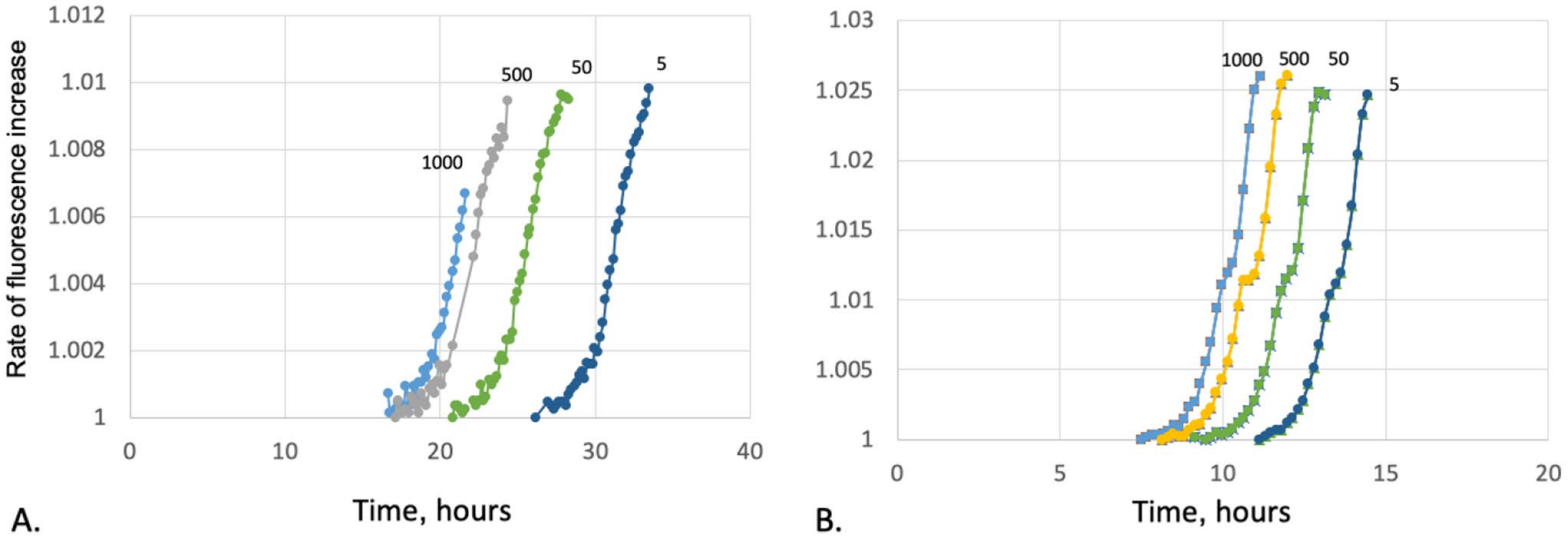
Rate of fluorescence increase (calculated as fluorescence of time point 2/time point 1) as a function of incubation time in hours for 4 different initial bacterial load-1000, 500, 50 and 5 Colony-forming units/mL. **A**. *S.aureus*, **B**. *E.coli*

Overall, the mean TTP was 15 h for *S. aureus* and 10 h for *E. coli*. As compared with the reference curves, this correlates with an approximate bacterial load of > 1000 CFU for *S. aureus* and 500–1000 for *E. coli* (Figs. 1 and 2). In cases of delay of > 12 h in the transfer of the culture to the laboratory, we adjusted the TTP to be 2–3 h shorter (for *S. aureus* and *E. coli*, respectively) but it did not change the bacterial load category in either case.

Overall, patients with *S. aureus* bacteremia were sicker. They had longer admissions, required vasopressors more frequently, and had higher disease severity scores (Table 4).

**Table 4:** Characteristics of patients with bacteremia. *N* = 60

Variable	*S. aureus*, *N* = 30	*E. coli*, *N* = 30	*p*
Mean age in years ± SD (range)	72 ± 16 (26,95)	76 ± 22 (19,98)	0.4
Male Gender, N(%)	16 (53)	13 (33)	0.6
Death, N(%)	15 (50)	8 (26)	0.1
Intensive care, N(%)	10 (33)	3 (10)	0.03
Bacteremia > 2 days, N(%)^*^	12 (44)	1 (3)	0.0001
Mean length of admission, days (range)	32 ± 31 (1,134)	11 ± 9.5 (1,35)	0.001
Cardiovascular morbidity, N(%)	20(66)	17(28)	0.5
Hypertension, N(%)	23(76)	19(63)	0.2
Diabetes, N(%)	20(66)	14(46)	0.1
Malignancy, N(%)	4(13)	10(33)	0.1
Immunosuppression, N(%)	3 (10)	6(20)	0.4
Dialysis, N(%)	7(23)	0	0.01
Mechanical ventilation, N(%)	9(30)	2(7)	0.04
Vasopressors therapy, N(%)	17(56)	4(13)	0.0001
Mean qSOFA score	1.5 ± 2 (1,3)	0.5 ± 1 (1,3)	0.01
Mean Pitt bacteremia score	3.5 ± 4 (0,12)	1 ± 2 (0,8)	0.01

Three patients died within 1 day of bacteremia, therefore the denominator was 27

**Fig. 3:**
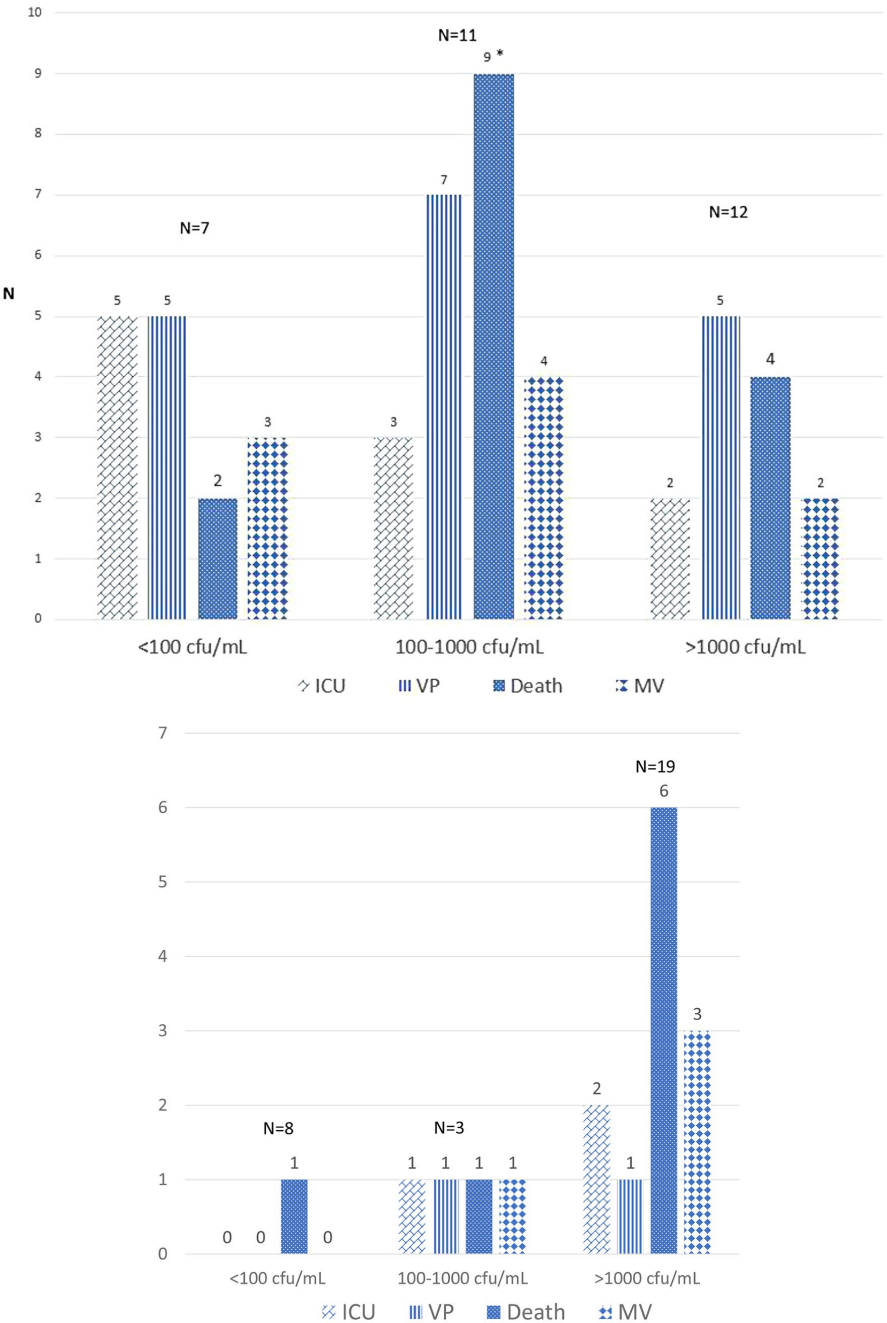
Number of patients who died, required intensive care (ICU), vasopressors (VP), or mechanical ventilation (MV) across the quartiles of bacterial inoculum in the blood in A. *S. aureus* and B. *E. coli* bacteremia. *p = 0.02

We compared clinical outcome across 3 bacterial load categories (Fig. 3). For *S. aureus* bacteremia, among patients with a bacterial load of 100–1000 CFU, death was significantly higher (OR for death = 9.7, 95%CI:1.6–59, *p* = 0.01). The results did not change with adjustment for methicillin-resistant *S. aureus* (MRSA). The Pitt bacteremia score was lower in the low bacterial load category: 2 in patients with < 100 CFU vs. 4 in patients with higher load, *p* = 0.04. Other characteristics of clinical severity did not differ between the bacterial load categories.

Endovascular infection with *S. aureus* was associated with a higher bacterial load (6/9 patients with endovascular infection and 5/21 without had bacterial load > 1000 CFU, *p* = 0.03). Bacterial load > 1000 CFU had an odds ratio of 6.4 (95%CI:1.2–35, *p* = 0.03) to predict an endovascular source. For *E. coli* bacteremia, the bacterial load was similar for urinary source vs. bile or unknown source. The presence of a foreign body (central venous or urinary catheter, pacemaker, mechanical valve, or prosthetic joint) was not associated with a significantly higher bacterial load for either pathogen. Except for 1 patient, *E. coli* was not associated with persistent bacteremia. For 12 patients with *S. aureus* bacteremia > 2 days, the initial bacterial load (in the culture at day 1) was not associated with the length of bacteremia.

## Discussion

In this study, we used the growth and detection plot graph (GDPG) created by the automated blood culture system to estimate the initial bacterial load in patients with *S. aureus* and *E. coli* bacteremia. First, we show that TTP is directly correlated with the initial inoculum. The bacterial growth curve is influenced by the growth rate, the initial bacterial load and the lag phase of growth [7]. This means that given the same growth rate, a long lag time or a small bacterial load can result in an identical growth curve. Since there is a direct correlation between TTP and the initial inoculum, the lag phase is assumed to be non-significant and we can estimate the initial bacterial load from the GDPG, retrieved from the BACTEC™ FX database.

Although the blood culture system is robust and supports bacterial growth even in suboptimal conditions [10], the bacteria grew in both the aerobic and anaerobic bottles in only 20–30% of cultures. This could not be explained by major differences in blood volume inoculated to the bottles. The preferential growth of *S. aureus* in aerobic and *E. coli* in anaerobic bottles suggests that the growth preferences of specific bacterial species influence the differential growth. Overall, the blood volume obtained in most of the blood cultures was suboptimal. Studies have reported low volume in up to 80% of blood cultures [11, 12]. It was estimated that each additional mL of blood collected can result in a 2–4% increase in positivity rate [11]. The mean blood volume obtained did not differ between cultures that grew *S. aureus* and those with *E. coli.*

We found that *S. aureus* growth was sensitive to the blood volume obtained whereas *E. coli* growth was less so. This could be explained by the faster growth of *E. coli* that “overcomes” the effect of sub-optimal blood volume. Additionally, a 12-hour delay in sample transfer to the microbiology laboratory resulted in a decrease in TTP by 2–3 h. Taken together, these findings reinforce the importance of obtaining a full set of culture bottles, with sufficient volume and prompt transfer to processing. Suboptimal handling of blood cultures and suboptimal blood volume seem to have a differential impact on culture results depending on the bacterial species, with the slower-growing *S. aureus* more sensitive than the rapidly growing *E. coli*.

Next, we estimated the bacterial load in the patients’ blood and compared it with clinical outcome. Patients with *S. aureus* bacteremia were sicker compared with patients with *E. coli* bacteremia. Their mean estimated bacterial load was higher (> 1000 CFU/mL, approximate TTP = 15 h), and the high bacterial load was associated with a 6-fold increase in the chance for an endovascular source for the infection. Moreover, patients with *S. aureus* bacterial load > 100 CFU/mL had a higher Pitt bacteremia score and a higher mortality rate. In line with our findings, a previous study by Khatib et al. found that time to positivity of ≤ 14 h was an independent predictor of an endovascular source of *S. aureus*, as well as associated with extended bacteremia, metastatic infection, and attributable mortality [3]. Another study showed that a TTP cut-off of 13 h had a 100% sensitivity to diagnose infective endocarditis [4].

Although *E. coli* grows faster than *S. aureus*, its mean bacterial load in the culture bottles was lower and patients were less sick. We did not find any correlation between high bacterial load of *E. coli* with multiple surrogate markers of disease severity. Moreover, to the best of our knowledge, such an association between *E. coli* bacterial load in the blood and poor clinical outcome was never clearly demonstrated in the literature.

In severe bacterial infections, various bacterial components, such as endotoxins, teichoic/lipoteichoic acid, peptidoglycans, and bacterial DNA, induce or enhance inflammation and may also be directly toxic to human cells [13]. We suggest that the dynamics of bacterial toxins is different between *S. aureus* and *E. coli* infections, and not always directly associated with the bacterial load. For example, in a rabbit model of MRSA necrotizing pneumonia, treatment with linezolid, but not with vancomycin, was associated with improved survival, a decrease in bacterial counts, and a parallel decrease in bacterial toxins and cytokines [14]. On the other hand, in a mouse model of sepsis (induced by cecal ligation), a high dose of imipenem was associated with a reduction of bacterial counts, along with increasing endotoxin and inflammatory cytokine levels. A lower dose of imipenem was associated with lower cytokine levels and better survival [15].

Our study has several limitations. First, although we obtained the first positive blood culture and verified that the patients were not treated with oral antibiotics before arrival, we cannot rule out that some patients were indeed partially treated. Second, the “standard” curves to which we compared the clinical cultures were constructed by injecting a known number of bacteria in broth into the culture bottles. Human blood may have different impact on bacterial growth compared with broth. Finally, our study was powered to detect differences in length of stay, which could lead to type II error.

In summary, the GDPG retrieved from the automated blood culture system can be used to estimate the initial bacterial load in patients with bacteremia. We found that the bacterial load was associated with worse clinical outcomes in *S. aureus* but not in *E. coli* bacteremia. Given the different growth characteristics between *S. aureus* and *E. coli* in the culture bottles, future research may examine the utility of the growth curves, as retrieved from the automated system, for faster identification of bacterial species or contaminants based on the shape of their curves.

## Electronic supplementary material

Below is the link to the electronic supplementary material.

Supplementary Material 1

Supplementary Material 2

## Data Availability

No datasets were generated or analysed during the current study.
