# Supplementary figures and images for: Estimating bacterial load in S. aureus and E. coli bacteremia using bacterial growth graph from the continuous monitoring blood culture system
Source: Eur J Clin Microbiol Infect Dis. 2024 Jul 29;43(10):1931–8. doi: 10.1007/s10096-024-04893-w (PMC11405537; doi:10.1007/s10096-024-04893-w)

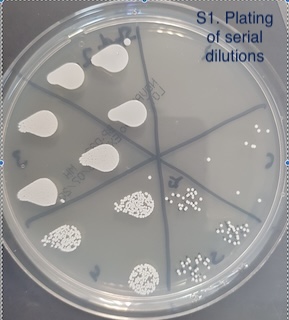

Supplement: Supplementary file 1 — Supplementary Material 1 [file 10096_2024_4893_MOESM1_ESM.jpg]
